# An Open-Source, Low-Cost Solution for 3D Scanning

**DOI:** 10.3390/s26010322

**Published:** 2026-01-04

**Authors:** Andrei Mateescu, Ioana Livia Stefan, Silviu Raileanu, Ioan Stefan Sacala

**Affiliations:** 1 Automatic Control and Systems Engineering Department, Faculty of Automatic Control and Computers, National University of Science and Technology Politehnica of Bucharest, Splaiul Independentei, No. 313, 060042 Bucharest, Romania; ioana_livia.stefan@upb.ro (I.L.S.);; 2 Automatics and Industrial Informatics Department, Faculty of Automatic Control and Computers, National University of Science and Technology Politehnica of Bucharest, Splaiul Independentei, No. 313, 060042 Bucharest, Romania

**Keywords:** 3D scanner, computer vision, open-source, linear laser

## Abstract

With new applications continuously emerging in the fields of manufacturing, quality control and inspection, the need to develop three-dimensional (3D) scanning solutions suitable for industrial environments increases. 3D scanning is the process of analyzing one or more objects in order to convert and store the object’s features in a digital format. Due to the increased costs of industrial 3D scanning solutions, this paper proposes an open-source, low-cost architecture for obtaining a 3D model that can be used in manufacturing, which involves a linear laser beam that is swept across the object via a rotating mirror, and a camera that grabs images, to further be used to extract the dimensions of the object through a technique inspired by laser triangulation. The 3D models for several objects are obtained, analyzed and compared to the dimensions of their respective real-world counterparts. For the tested objects, the proposed system yields a maximum mean height error of 2.56 mm, a maximum mean length error of 1.48 mm and a maximum mean width error of 1.30 mm on the raw point cloud and a scanning time of ∼4 s per laser line. Finally, a few observations and ways to improve the proposed solution are mentioned.

## 1. Introduction

In a future where almost every task a worker may undertake in a factory has been automated by humans with the introduction of robots and machines, a few tasks are yet to be automated. These jobs present difficulties in automation because they need a high level of dexterity or must be completed in unstructured environments, which makes use of both two-dimensional (2D) and 3D artificial vision (AV).

Robots conduct the majority of their tasks “blindfolded”, in the sense that they largely depend on exact timing and positioning, with next to no information on the structure of the environment. This represents the largest challenge of the robotics industry at the moment. Because of their eyesight and powerful brains, humans are able to perform tasks, like object sorting, with ease and therefore, until the vision barrier is breached, a robot cannot simply surpass and replace every human, despite its speed, dexterity, precision, and force [1].

The process of analyzing one or more objects to convert and save their features—such as size, shape, and even color—in a digital format is known as 3D scanning. The result of the scanning process, saved as a point cloud or a mesh, is used to generate a 3D model, with applications in various domains, like quality control and inspection in industrial environments, vision-guided robot systems, the entertainment industry, such as movies, games and virtual reality (VR), motion capture (MoCap), design, reverse engineering, orthotics and prosthetics, and the digitization of cultural artifacts [2].

For obtaining such a model, multiple types of devices called 3D scanners, each with its own advantages, disadvantages, and limitations, can be used. To begin with, these can be classified as “contact scanners”, like a Coordinate Measuring Machine (CMM), which examines the object’s surface through physical touch, using a probe mounted on a robotic arm that is moving on the surface of the object, scanning it by measuring the coordinates of the points that the probe is passing through, and “non-contact scanners” that do not require direct contact with the object, but instead rely on using some sort of radiation in order to analyze the 3D object.

When using contact scanners, the objects must be fixed on the surface they are sitting on, in order to stay stationary while the scanning is in progress. Furthermore, the physical touching of the object can lead to its deterioration, and the scanning process is also slower compared to non-contact scanning, while having the advantage of high accuracy, which is one of the reasons this type of scanning is used in inspection and quality assurance.

Regarding the type of radiation, non-contact scanners, on which we will focus in this work, can be divided into passive scanners, which do not emit any type of radiation, but instead use the radiation already present in the environment, like visible ambient light (e.g., Computer Stereo-Vision that uses two digital video cameras as sensors, slightly apart from each other (like human eyes), “looking” in the same direction), and active scanners, which use their own source of radiation, such as light, ultrasound, and X-ray, to perform the scanning of the object.

One example of an active scanner is the Structured Light 3D Scanner, which projects a structured pattern of light onto the object, such as parallel grids or some common geometric patterns, and then uses a camera placed at an angle from the source of light to take pictures of the deformed pattern.

Another non-contact active scanning method of acquiring 3D point cloud representations of objects is by utilizing lens-free sixth generation (6G) wireless sensing solutions [3], reliable only for larger, less complex objects, but with the advantage of seeing through walls and foliage, and working in all illumination scenarios.

A Linear Laser 3D Scanner, also called a Triangulation-Based 3D Laser Scanner, is a type of scanner that launches a laser line onto the surface of the object, and captures the scene imagery on a camera, to then compute the distance to the subject. The term “triangulation” refers to the triangle made by the laser, the camera, and the object.

Given the distance from the laser to the object, the distance between the camera and the laser, and also the camera angle being known, the shape and size of the triangle can be precisely determined. The size of the triangle is mandatory in order to obtain the distance by processing the images captured by the digital camera. This type of laser scanner allows the scanning of a single object and even of a multitude of objects at the same time (for example, objects placed in a bin that needs sorting) [4].

Scanning can be performed in two ways, depending on the configuration of the laser and of the scene: fixed laser with moving scene, and moving laser with fixed scene. The first option involves the fixed positioning of the laser and a method for moving the object/objects through the linear beam projected on them, by a conveyor belt or with a rotating platform, for example. The second alternative implies moving the laser and the camera over the scene, while maintaining the objects’ stationary position.

Active scanners can be easily influenced by ambient light, which must be eliminated or reduced as much as possible. Transparent and reflective surfaces can prove difficult to digitize with a limited range and number of possible applications. Precision of this system is given by the camera resolution and the step of the conveyor belt/rotating platform/laser and camera. In the case of continuous motion, the speed of the moving conveyor belt and the recording speed of the camera, measured in frames per second (FPS), influences the quality of the model. A camera calibration is also needed to obtain the pixel-to-mm ratio.

## 2. State of the Art

### 2.1. 3D Scanning Applications in Various Industry Sectors

Three-dimensional scanning enables precise and efficient digital representation of real-world objects and has become a vital technology for modern manufacturing and beyond. By capturing detailed surface geometry, 3D scanners [5,6,7,8] create accurate virtual representations that can be used across industries, for quality control, reverse engineering, and rapid prototyping, significantly improving design accuracy and production speed.

These technologies can be leveraged for extracting the features of objects. For example, paper [9] proposes a method for computing the volume of irregularly shaped 3D objects, combining structured-light 3D scanning (performed using an HP 3D Structured Light Scanner Pro S3) with MATLAB tools and algorithms. This method was validated on non-uniform objects, namely limpet seashells, turning the 3D scans into accurate digital models. They demonstrated that data gathered through structured-light 3D scanning can be utilized to obtain accurate and reliable volume measurements of irregularly shaped objects.

For small and complex parts with multiple holes, an automatic 3D scanning system was developed in paper [10]. The system uses the design model of the part to plan scanning paths, capture all features accurately, and produce a measured 3D model. When tested, this method provided higher efficiency and accuracy compared to traditional manual or semi-automatic scanning methods, especially for small, complex parts.

In [11], an in-line inspection system for Fused Deposition Modeling (FDM) 3D printers was developed, providing the means for scanning and measuring parts without the need for contact, while they are being printed, to detect geometric errors in real time, establishing the foundation for developing AI-driven self-correcting 3D printing systems. While some practical challenges remain (such as surface reflection and speed trade-offs), the scanning system was able to accurately measure the objects during printing and to detect deviations in real time, proving its feasibility, accuracy, and potential for advancing adaptive, closed-loop 3D printing systems.

In paper [12], a product inspection station was developed for industrial quality control, which combines 3D scanning together with cloud-based analysis and remote human inspection to monitor the process. The paper represents a step forward towards fully automated quality control for Industry 4.0 manufacturing.

In the medical field, 3D laser scanning facilitates the creation of custom prosthetics, implants, and anatomical models, enhancing personalized healthcare and surgical planning. For example, paper [13] presents a technical review on the current clinical and medical applications of 3D laser scanning technologies. Their comprehensive analysis encompassed the main 3D body surface scanning technologies, namely laser triangulation, structured light, time of flight, and photogrammetry, and looked into their clinical applications, strengths, and weaknesses. This paper represents a guide for healthcare professionals in selecting suitable scanners for medical use, while it also highlights the research opportunities for improving 3D body scanning in medicine.

Similarly, in forensic science, 3D laser scanning can be used to document crime scenes and accident reconstructions with spatial detail, preserving crucial evidence for investigation and courtroom analysis. Paper [14] consists of a forensic case study demonstrating how 3D laser scanning technology, specifically the Trimble X7 3D laser scanner, can be used to document and analyze real-world crime and accident scenes with high precision. By capturing detailed 3D digital models of the scenes, they showed that 3D laser scanning improves documentation, analysis, and evidence reliability, reinforcing its importance in the growing field of 3D Forensics (3DFS).

The education field represents another important opportunity for 3D scanning solutions, where the ability to create 3D models of objects can be harnessed, as seen in [15,16]. In paper [15], an EinScan HX 3D scanner was used to digitally capture and model plants, creating detailed 3D virtual representations of different plant species. They used both rapid and laser scanning methods and compared the techniques, obtaining high-quality 3D virtual models for a digital plant library. The study demonstrated the feasibility, reliability, and importance of this approach for research, design, and education applications.

Therefore, while particularly important for manufacturing, 3D scanning provides the means for bridging the physical and digital worlds by modernizing, increasing precision, and improving efficiency across diverse industries. This opens the path for transferring and integrating the latest 3D scanning innovations, initially conceived for manufacturing, to a plethora of other research fields.

### 2.2. Low-Cost 3D Scanning

There are many manufacturing 3D scanning-based solutions for bin picking [17,18,19,20], while trajectory generation is also intensely researched [21,22,23], with industrial solutions available [24] and more specific applications such as cutting and welding tasks [25,26].

While industrial products offering solutions for 3D scanning-related tasks exist, their main disadvantage is the high costs. Therefore, developing open-source solutions ensures that people can easily access the know-how and can use it to implement the methods themselves, therefore proving transparency and implementation flexibility, while keeping the costs low.

Paper [27] investigates a developed low-cost 3D scanner in the manufacturing setting and compares it to a commercially available solution (Faro Focus S150) in terms of depth resolution, scanning distance and point density. The prototype is based on Microsoft Kinect V2, MATLAB, and a custom hardware platform, with a cost of USD 500 (without the costs of software and licenses), while noting that the prices of the solutions available on the market start at ten thousand USD. The Faro Focus S150 has a depth resolution of 0.3 mm at a distance of 10 m, with a 90% reflectivity, while the Microsoft Kinect solution has a lower depth resolution of approximately 3 mm at a distance of 3 m.

Paper [28] consists of a survey on low-cost 3D laser scanning technology, based on moving 2D LiDAR to obtain 3D maps of the environment economically, analyzing solutions with prices of up to USD 306 and noting the limitations of the technology (such as compromising on the real-time performance and challenges brought by moving objects in the environment).

A low-cost 3D scanning setup was implemented in paper [29] to create meshes of objects using an open-source software for 3D scanning and 3D mesh processing. For capturing the object surface, they used a hand-held red laser line (RM 12) and applied triangulation techniques. They applied Screened Poisson Surface Reconstruction techniques to filter the data and obtain a smooth mesh. While their implementation is not completely transparent, the obtained mesh measurement error was between 5.01% and 9.39% on their test cases (four objects of different colors).

## 3. Hardware Structure and the Concept of Triangulation

In this work, an open-source and inexpensive triangulation-based solution for 3D scanning is proposed. The source files and the scanning results present in this work are available on the GitLab repository at https://gitlab.cs.pub.ro/andrei.mateescu0612/3d-scanner (accessed on 31 December 2025), where *Control_stepper_USB.ino* is the code that runs on the Arduino UNO board, *scanning_procedure.py* is the code that runs on the Raspberry Pi board, *serial_communication example.py* is a code example for controlling the stepper motor from the Raspberry Pi board, through the Arduino UNO board, compatible with the code in *Control_stepper_USB.ino*, and in *Scanning results* are the scanning results presented in Section 6 of the paper, as point clouds.

Starting from this section, several notations will be used for explaining the inner workings of the proposed 3D scanner, which can be observed in Table 1.

**Figure 1:**
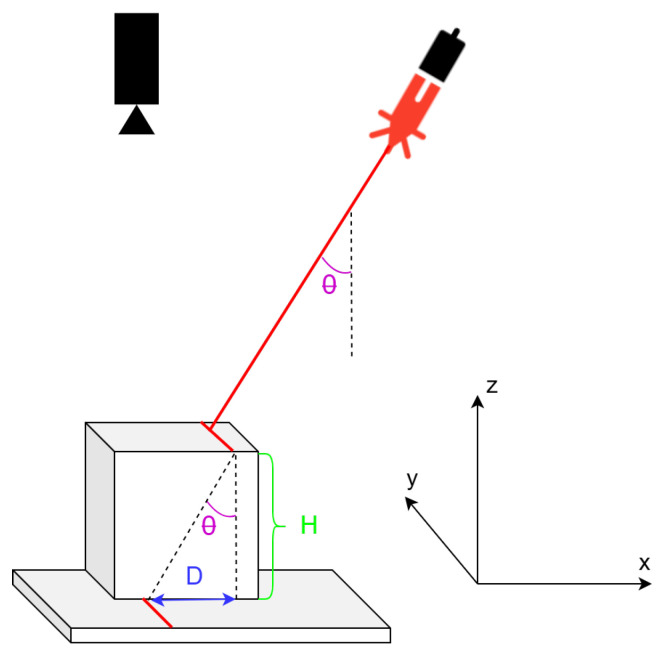
The concept of triangulation scanning.

The architecture for this system is displayed in Figure 1. The laser line needs to pass over the object from one side to the other, and the camera takes pictures after the laser line has moved by a certain amount. The height *H* of the object (z-axis) is determined using the information extracted from the image, which is the distance *D*.

In the triangle, it can be observed that

(1) $$tan\left(\theta \right)=\frac{D}{H}$$

or, equivalently,

(2) $$H=\frac{D}{tan(\theta )}$$

**Figure 2:**
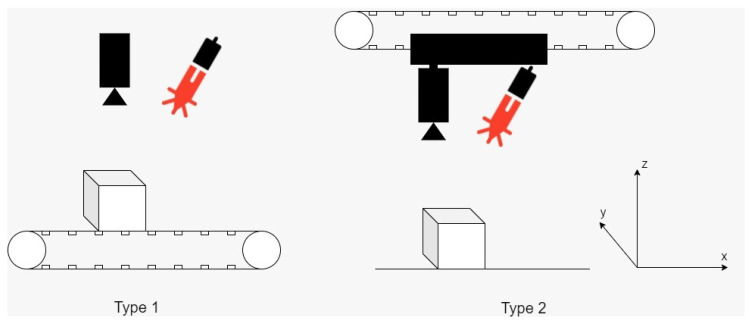
Different types of hardware configurations.

There are different ways to move the laser line from one side of the object to the other, and depending on this, the components can be mounted in several configurations, like in Figure 2:

The first design (Figure 2, Type 1) for the hardware implementation is a fixed laser and camera with a moving scene (the cube placed on the conveyor belt), while the second type (Figure 2, Type 2) is a moving laser and camera with a fixed scene (the laser and camera ensemble is mounted on a conveyor-type mechanism that allows movement on a one-dimensional (1D) axis, the object being stationary on the floor). While this method might work in theory similarly to the first type, the disturbances induced by the movement of the camera and laser could result in poor scanning quality. In these two scenarios, the controller will measure the position of the conveyor belt using encoders and link this information with the one from the camera, regarding the height of the object.

**Figure 3:**
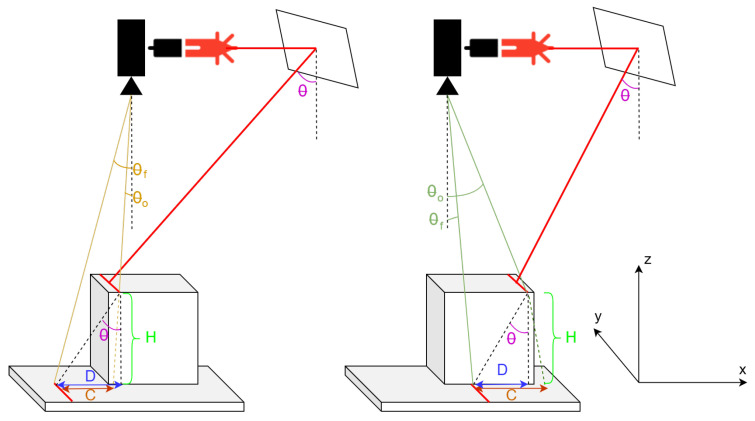
Diagram displaying the chosen system architecture.

The proposed solution implies the use of a third type of configuration, in which the camera, the laser and the scene are stationary, but the laser beam is reflected onto the scanned surface using a mirror, which rotates around its center as seen in Figure 3.

This configuration reduces the complexity of the system and the appearance of disturbances while improving the precision. In this case, the θ angle is the one that changes. The object height will be obtained using the triangulation principle, as described earlier in this section, but with a small twist. It can be noticed that in this scenario, the distance seen by the camera is not the distance *D*, but another one denoted *C*. From the image taken by the camera, beside the distance *C* (computed in pixels and later transformed into mm), the angles $\theta_{o}$, for the laser line that falls on the object, and $\theta_{f}$, for the laser line that falls on the floor, are also computed.

**Figure 4:**
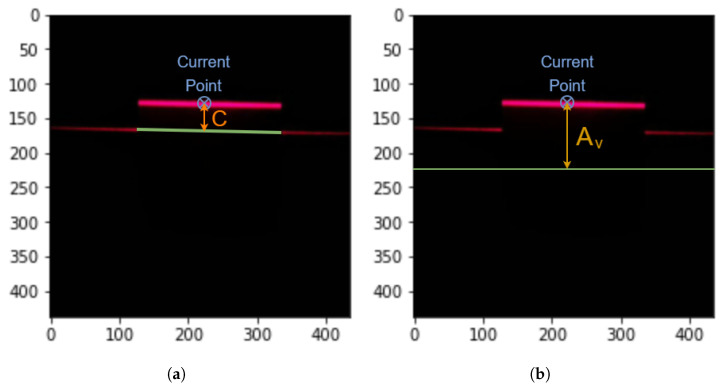
Distance *C* (**a**) and distance $A_{v}$ (**b**) obtained from the image.

In Figure 4a, the distance *C* is obtained from the image as the distance between the laser line that falls on the floor (represented here as the green line) and the point on the object, and in Figure 4b, the vertical distance $A_{v}$ is obtained as the deviation from the middle of the image (the horizontal green line, where $\theta_{o}$ is 0 degrees) and the respective point, which is used to compute the angle $\theta_{o}$ as in Equation (3), where $H_{cam}$ is the camera height. For such an image, this operation is performed for every pixel that represents the laser line reflecting from the object.

(3) $$\theta_{o}=arctan\left(\frac{A_{v}}{H_{cam}}\right)$$

Figure 3 illustrates the relations between the θ and $\theta_{o}$ angles, and the *D*, *C* and *H* distances, which have been represented in Equations (4) and (5), and if Equation (4) is substituted in Equation (5), then Equation (6) is obtained.

(4) $$tan\left(\theta \right)=\frac{D}{H}$$

(5) $$tan\left(\theta_{o}\right)=\frac{D-C}{H}$$

(6) $$tan\left(\theta_{o}\right)=\frac{H\cdot tan(\theta )-C}{H}$$

Equation (6) can be rewritten as

(7) $$H\cdot tan\left(\theta_{o}\right)=H\cdot tan\left(\theta \right)-C$$

Given that the θ angle is known, being measured when the mirror rotates and the laser line moves, and knowing the aforementioned values, the height of the object, *H*, can now be determined as

(8) $$H=\frac{C}{tan\left(\theta \right)-tan\left(\theta_{o}\right)}$$

Finally, substituting Equation (3) in Equation (8), the height of the object can be written in the following, more compact and computationally efficient form,

(9) $$H=\frac{C}{tan\left(\theta \right)-\frac{A_{v}}{H_{cam}}}$$

## 4. Hardware Implementation

**Figure 5:**
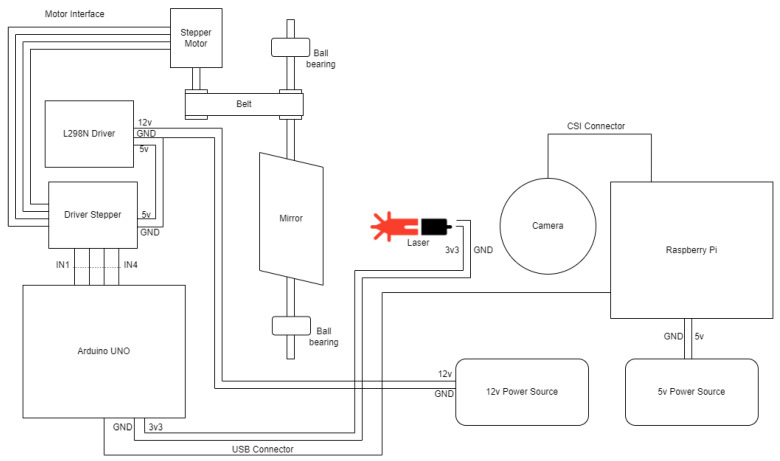
The hardware layout.

The top-down layout of the architecture can be seen in Figure 5, including the hardware components needed for the implementation, mounted on a support structure. The Raspberry Pi is powered by a 5 V Direct Current (DC) source through a Mini Universal Serial Bus (USB) cable and sends commands to an Arduino UNO-compatible board (using the serial interface), which controls the tilt (angle) of the mirror via a stepper motor.

The command sent to the Arduino UNO board represents the number of steps the motor rotates and the direction of rotation. The stepper motor is controlled by a stepper motor driver, powered by a 5 V DC converted from a 12 V current by an L298N driver. The motion is transmitted from the stepper motor’s output shaft to the mirror’s shaft through a toothed belt and two toothed pulleys. The mirror is fixed and free to rotate due to two ball bearings, one on either side, which are mounted on the structure using two brackets. The laser, which is stationary, is also controlled by the Arduino UNO board to ensure that it activates only when needed. The mirror and laser calibration is performed by placing a target behind the laser, at the same level as the mirror and laser, and positioning the mirror so that the reflected laser line hits the target precisely on its center. By performing this procedure, the θ angle is initially set at 90 degrees.

After every repositioning of the laser beam (reflected by the mirror), the Raspberry PI commands the Raspberry Pi 2018 HD Camera V1.0 to take a picture to be further processed. After an image is processed, the data is saved and the image is discarded. Next, the laser line is moved again by one step, another image is grabbed and sent to the Raspberry PI to be processed, and the cycle is repeated.

**Figure 6:**
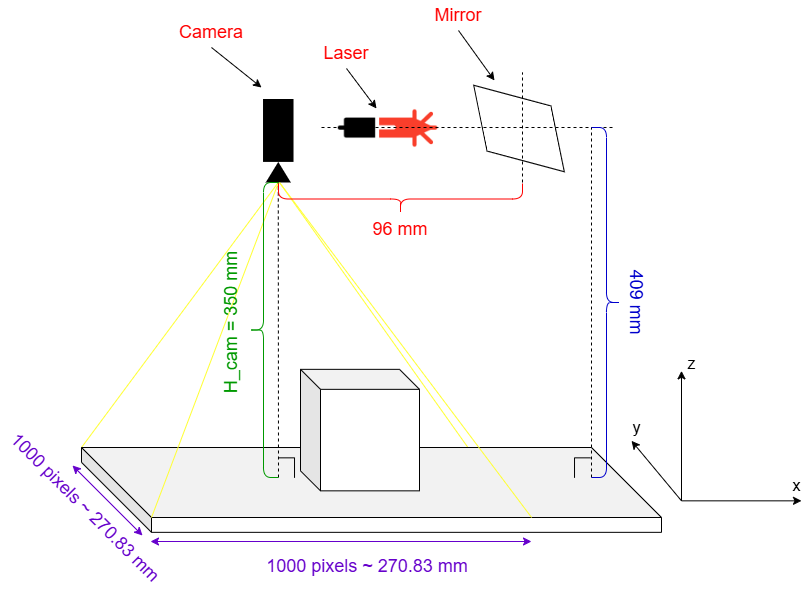
The hardware dimensions.

A list of hardware parameters and their values can be visualized in Table 2, including the camera height, camera resolution, camera field of view and stepper motor parameters. In this work, the stepper motor was used in full stepping mode, but the scanning resolution, i.e., the number of laser lines for a full scan, can be doubled when used in the half-stepping mode. The stepper motor reduction gear set introduces about 30 steps of backlash in the system, or approximately 5 degrees, but this represents no problem since the mirror only rotates in one direction during a scanning procedure, and then it can reset to the original position by completing the full turn. The presented dimensions can also be visualized in Figure 6.

**Figure 7:**
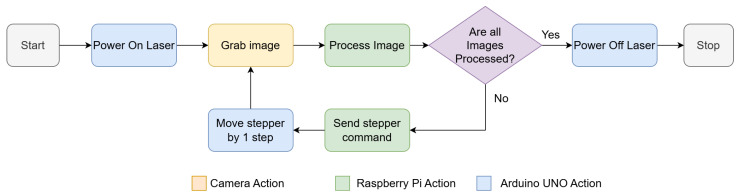
The machine state diagram.

A diagram containing the same series of events is presented in Figure 7, and the algorithm for the scanning procedure can be visualized in Algorithm 1. For the current size of the structure, up to 100 laser lines can be obtained for one scanning process, equivalent to 100 images. Throughout the experiments, it has been observed that the duration for moving the laser, taking the picture (1000 × 1000 pixels), processing it and saving the data is roughly 4 s, and this amounts to a total time of about 400 s for scanning the entire visible surface.
**Algorithm 1** The pseudo-code algorithm for the scanning procedureget_camera_calibration(calibration_images)     ▹ Used to undistort the captured imagesconnect_to_arduino()                ▹ To control the stepper motorN←number_of_images          ▹ The number of images is set by the useri←1**while** i≤N **do**   image←capture_image()   image←undistort_image(image)   points←process_image(image)   append_to_file(points)   move_laser()                 ▹ The laser is moved by one step   i←i+1**end while**close_connection_to_arduino()

**Figure 8:**
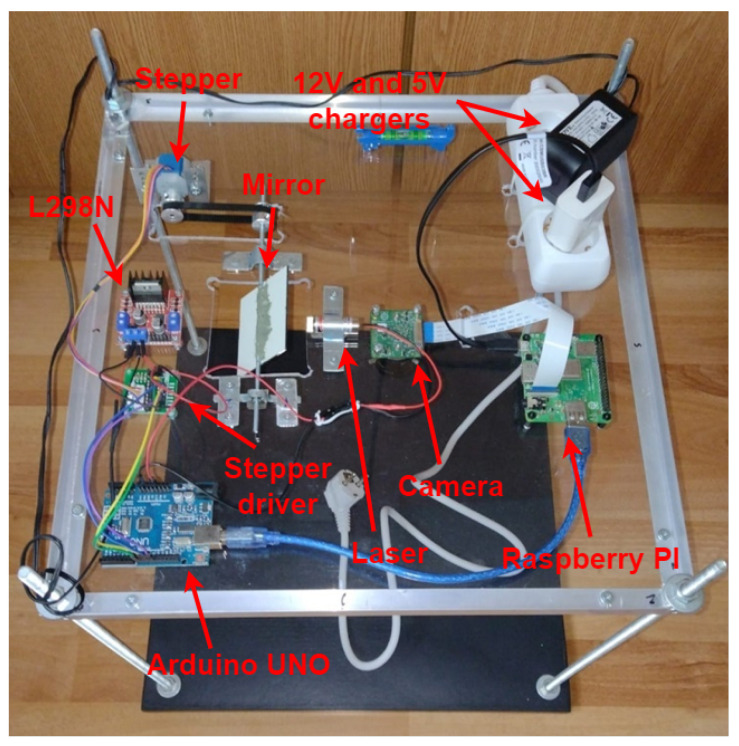
The hardware structure top-down view.

**Figure 9:**
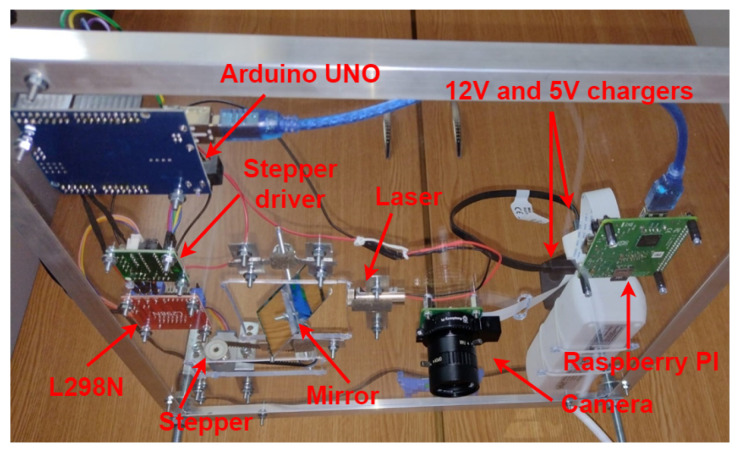
The hardware structure, bottom-up view.

The resulting structure can be visualized in Figure 8, from a top-down perspective, and in Figure 9, from a bottom-up perspective, with red labels and arrows highlighting each component.

To further demonstrate the low-cost aspect of the implementation, a cost analysis was performed, and the resulting cost of the equipment was compared with that of an industrial solution.

A Raspberry Pi 4 board, similar to the one used, can be found online at around USD 70 [30,31] and a Raspberry Pi camera is under USD 30 [32,33], while a UNO-compatible board is under USD 10 [34,35] and a 28BYJ-48 stepper motor and ULN2003 driver (like the ones used in this implementation) are near USD 3 [36,37]. Finally, a linear laser module can be bought for under USD 2 [38,39], which brings the cost of the assembly to about USD 115.

Compared to an industrial solution, rated at around USD 10,000 brand new [40], or even pre-owned [41], the proposed solution is two orders of magnitude less expensive, cementing it as a low-cost alternative for 3D scanning.

## 5. Image Processing

**Figure 10:**
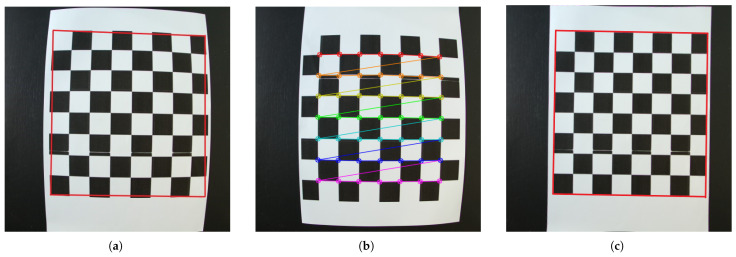
(**a**) The distorted image, (**b**) the identified chessboard pattern’s corners and (**c**) the corrected image, with the red frame highlighting the distortion.

From the start, a camera calibration procedure was needed to eliminate the barrel distortion introduced by the camera. This has been performed with the aid of the OpenCV library and a printed chessboard pattern, with the square size of 26 mm and a total of 4 × 4 black squares (with a total of 8 × 8 squares). A total of 17 images were obtained by moving and rotating the chessboard pattern on the entire camera field of view, the *findChessboardCorners* OpenCV method was applied to identify the pattern’s corners in the images, *calibrateCamera* to obtain the lens parameters that will be used for calibration, and *undistort* to undistort the images, resulting in a pixel-to-mm ratio of 0.27083 mm/pixel. The distorted before-image and the undistorted after-image can be seen in Figure 10a,c, as well as the identified chessboard pattern’s corners in Figure 10b.

To perform the image processing and obtain the height, width, and length of the scanned object, a script has been implemented in Python v3.11.9 that uses a picture of the laser line reflecting from the surface of an object as input and performs the following actions:

1. **Color thresholding, to obtain a binary image**

**Figure 11:**
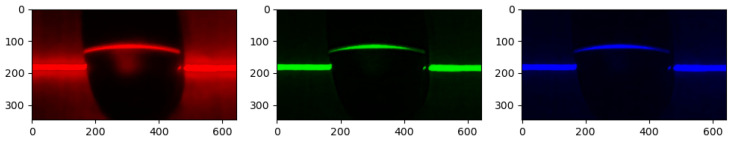
The three Red, Green and Blue (RGB) channels.

**Figure 12:**
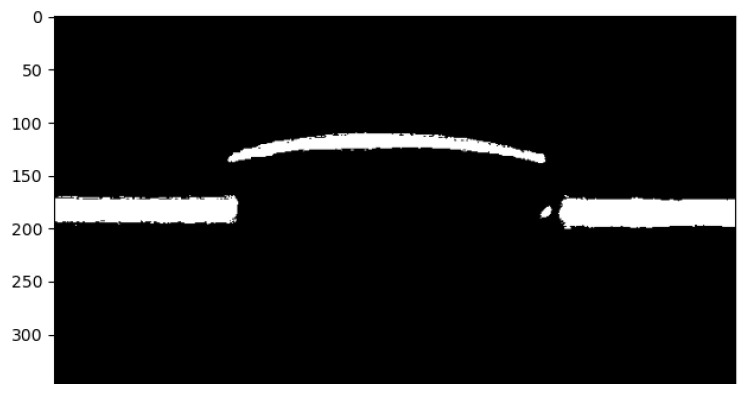
The resulting image after applying a threshold.

An example of a laser line reflected on the surface of a computer mouse can be observed in Figure 11 for every channel, where the red channel is the noisiest of them all, with the green being the least noisy (the cleanest) of them, while also lacking some information. The image has to be thresholded on every channel, keeping the pixels that have values above the specified thresholds, in this case the value of 150 on the red channel and 40 on the green and blue channels, to obtain the best result with the current illumination, and the result can be seen in Figure 12. This approach is very sensitive to ambient light intensity variations, and whenever the ambient light changes, the thresholds must be modified in a heuristic approach.

Another way for obtaining a binary image, in a more robust manner to ambient light variations, is by applying a relative threshold to the image, meaning that the pixels with values above this variable threshold are selected to form the binary image. The further described method can also be observed as a pseudo-code algorithm in Algorithm 2. Consider evaluating the values on columns, where the threshold can be selected, for example, as 90% of the maximum value for the red channel on the vertical axis, and applying it for all pixels on the respective column, and then repeating this operation for every column in the image. This operation yields similar results in most cases, but in some scenarios, where objects have lighter colors, or there is additional light in the environment, the extra noise present in the image is not affecting the operation as much.
**Algorithm 2** The pseudo-code algorithm for applying a variable thresholdimage←capture_image()thresholded_image←zeroes_like(image[:,:,0])    ▹ One channel zeroes array of image sizen←length(image[:,0,0]           ▹ Number of lines extracted from the imagem←length(image[0,:,0])        ▹ Number of columns extracted from the image**for** i←0 to *m* **do**   maximum←max(image[:,i,0])            ▹ Maximum value on column *i*   **for** j←0 to *n* **do**      **if** image[j,i,0]>threshold∗maximum **then**     ▹ Threshold chosen by the user       thresholded_image[j,i]←1      **end if**   **end for****end for**

2. **Thinning of the laser line, by averaging on the vertical axis**

**Figure 13:**
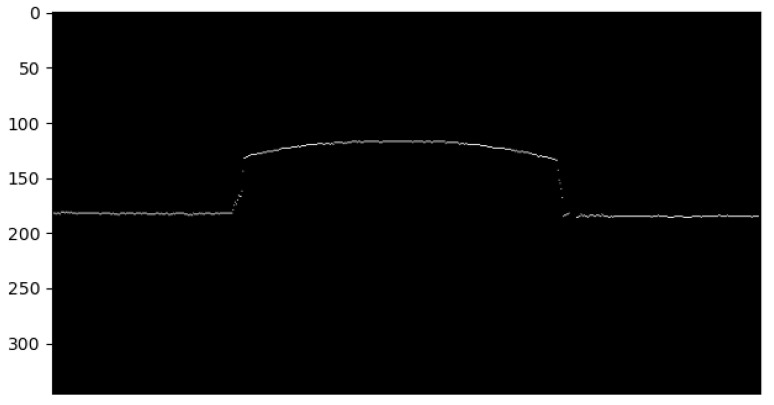
The result of the thinning process.

Due to the thickness of the line, some further processing was mandatory. In order to obtain a thin line, the average value on the vertical axis for the white pixels was computed. The result is present in Figure 13, with only one white pixel on each column. It should be noted that there might be some cases in which no pixel is present in some columns due to discontinuities in the laser line, as seen in the figure. In this case, the resulting point cloud will also be missing the respective points.

3. **Computing the distance $A_{v}$ in pixels**

The distance $A_{v}$ was obtained by subtracting the vertical coordinates of the points on the object from the vertical position of the middle line of the image, as presented in Figure 4b, and saving it in a 1D array.

4. **Calculating the distance *C* in pixels**

This step was accomplished by subtracting the minimum vertical value from the vertical value of every white pixel in the image and saving it in a 1D array.

5. **Applying a linear regression**

**Figure 14:**
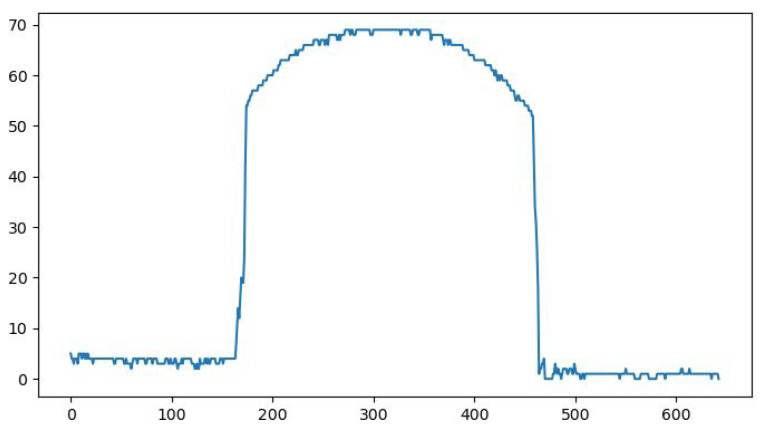
The first result of the distance *C* in pixels.

**Figure 15:**
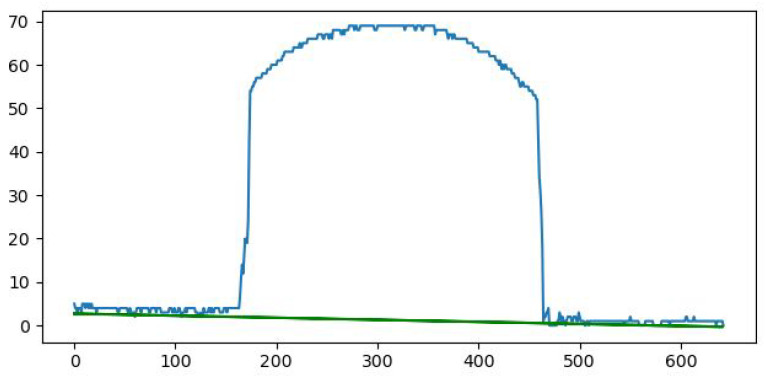
Height in pixels (blue) overlaid with the result from the linear regression (green).

**Figure 16:**
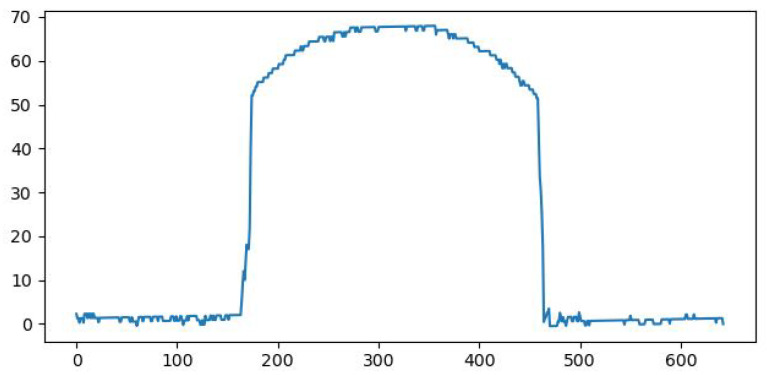
The corrected height for the points.

Looking closely at Figure 14, it can be observed that the obtained line is not perfectly horizontal; thus, a linear regression was needed. This step has been achieved using the laser points from the horizontal surface, and the before and after images can be seen in Figure 15 and Figure 16.

6. **Computing the height *H* in mm**

The distance C is now converted in mm with the aid of the pixel to mm ratio, the angle $\theta_{o}$ is computed using Equation (3), and then used accordingly to Equation (8) for obtaining the height of the object.

7. **Computing the width of the object (y-axis)**

**Figure 17:**
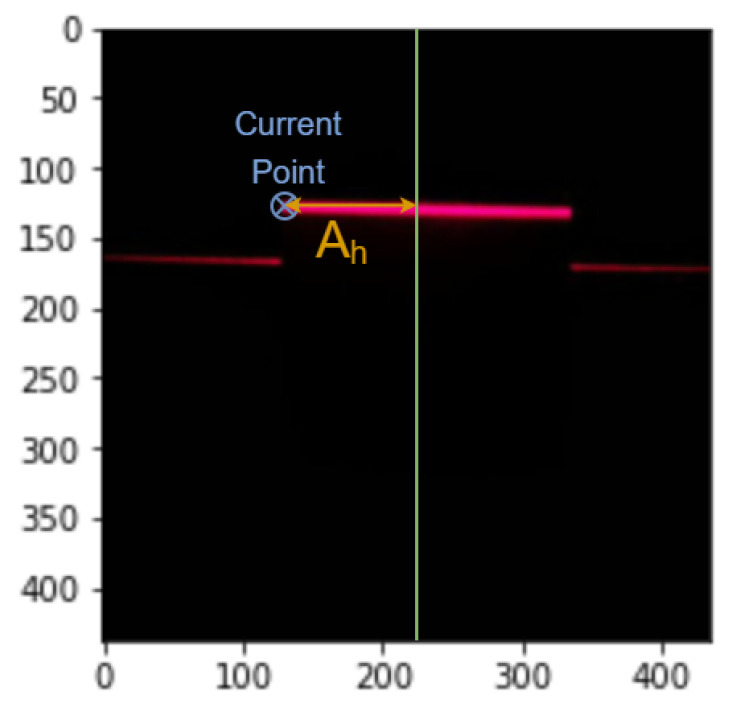
The distance $A_{h}$ obtained from the image.

**Figure 18:**
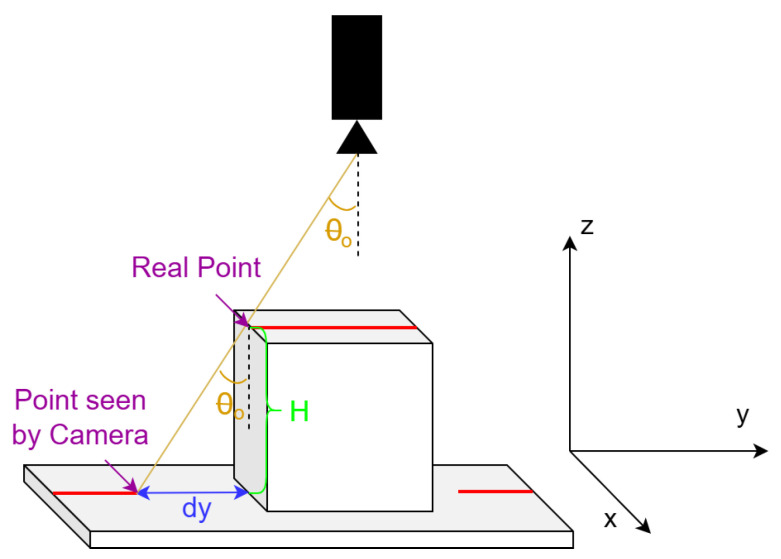
Obtaining the distance dy in order to compute the width (y-axis).

Considering the leftmost column to be at 0 mm and knowing the coordinates of the laser points reflected on the floor, each pixel of the laser line will be permuted according to its height. Firstly, the distance between the current point/pixel and the center of the image is computed, as explained in Figure 17, and then dy, which will be the distance to permute the point as seen in Figure 18, is calculated as in Equation (10), where $\theta_{o}$ is obtained from $A_{h}$ like in Equation (12). The real coordinate of the pixel on the y-axis is finally computed as the coordinate of the point on the floor $y_{floor}$, as seen by the camera, permuted by the distance dy.

(10) $$dy=H\cdot tan(\theta_{o})$$

(11) $$\theta_{o}=arctan\left(\frac{A_{h}}{H_{cam}}\right)$$

(12) $$y=y_{floor}+dy$$

8. **Computing the length of the object (x-axis)**

**Figure 19:**
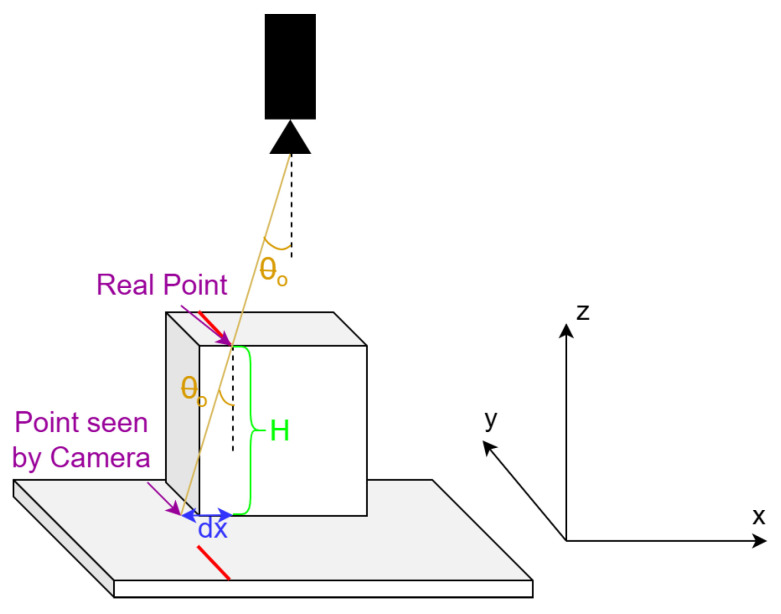
Obtaining the distance dx in order to compute the length (x-axis).

A similar strategy is applied for the length (Figure 19), as for the width, but in this case, the distance $A_{v}$ is used, as defined earlier, instead of $A_{h}$, yielding the following formula for dx:

(13) $$dx=H\cdot \frac{A_{v}}{H_{cam}}$$

and the following equation for the position on the x-axis:

(14) $$x=x_{floor}+dx$$

where $x_{floor}$ will be obtained as the product between the tangent of the laser angle and the mirror height, meaning that $x_{floor}$ will be null for the laser points directly beneath the mirror, when the laser line falls perpendicularly on the bottom surface.

## 6. Results

**Figure 20:**
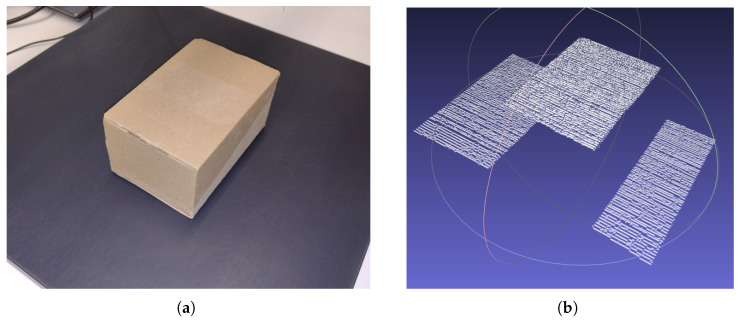
(**a**) The real box and (**b**) the point cloud resulted from the scanning process, visualized in MeshLab.

Four box-like objects have been scanned once, and in the following paragraphs, a comparison between the measured dimensions of the real object and the dimensions of the point cloud resulting from the scanning process is made. The result of a scanning procedure can be observed in Figure 20.

In Table 3, the real and scanned dimensions for the four scanned objects, named Box 1, Box 2, Box 3, and Box 4, have been structured and provided for visualization and comparison. For Box 1, 12 images have been used, resulting in 12 laser lines. For Box 2, a total of 30 images were captured. For Box 3, 64 images were grabbed, and finally, for Box 4, 39 images were utilized for computing the 39 resulting laser lines. For every axis, the minimum, maximum, and average dimensions are presented, along with the variance, mean error, and standard deviation (SD), as the difference between the measured dimensions and the real dimensions of the object.

It can be observed that the mean error for the height varies from the smallest absolute value of 0.55 mm in the case of Box 3 to the highest absolute value of 2.56 mm for Box 2, while the variance varies from the value of 0.43 mm for Box 1 to the value of 1.47 mm for Box 2.

The minimum length mean error is obtained for Box 2, with the value of 0.52 mm, while the maximum length mean error is obtained for Box 1 and has an absolute value of 1.48 mm. The variance for the length is considerably smaller compared to the height, with the minimum value of 0.004 mm for Box 1 and the maximum value of 0.104 for Box 2.

When looking at the width, it can be seen that the smallest mean error is obtained for Box 1, having the absolute value of 0.59 mm and the largest value is obtained when scanning Box 3, with a value of 1.30 mm. The width variances are smaller than the ones obtained for height, similar to the case of the length, the smallest variance obtained is of 0.017 mm for Box 1 and the largest variance of 0.44 mm for Box 4.

**Figure 21:**
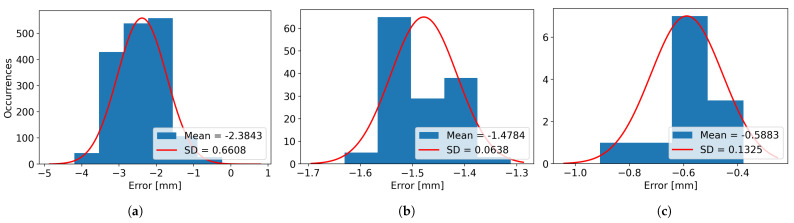
(**a**) The Height Error Histogram, (**b**) the Length Error Histogram and (**c**) the Width Error Histogram for Box 1.

**Figure 22:**
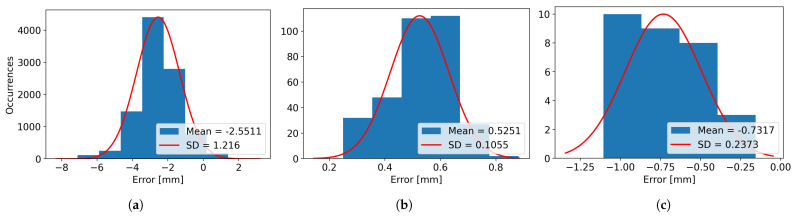
(**a**) The Height Error Histogram, (**b**) the Length Error Histogram and (**c**) the Width Error Histogram for Box 2.

**Figure 23:**
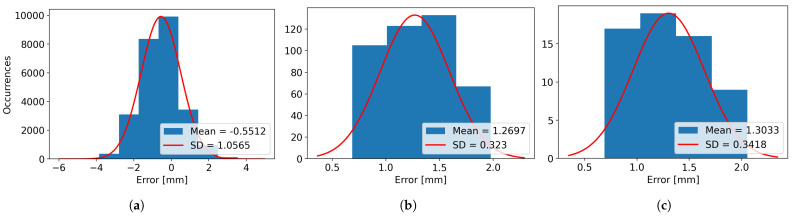
(**a**) The Height Error Histogram, (**b**) the Length Error Histogram and (**c**) the Width Error Histogram for Box 3.

**Figure 24:**
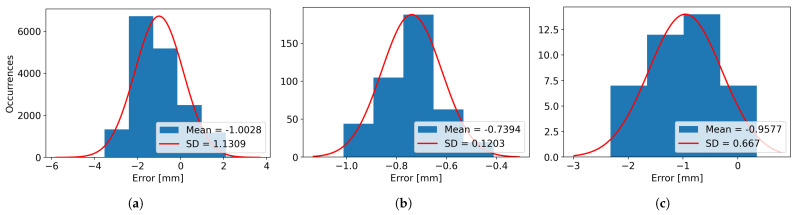
(**a**) The Height Error Histogram, (**b**) the Length Error Histogram and (**c**) the Width Error Histogram for Box 4.

The same results can be visualized as histograms, in Figure 21 for Box 1, Figure 22 for Box 2, Figure 23 for Box 3, and Figure 24 for Box 4, plotted for bin widths equal to the value of the SD in that histogram and where the mean values and the SDs (both in mm) can be observed in the figures’ labels.

**Figure 25:**
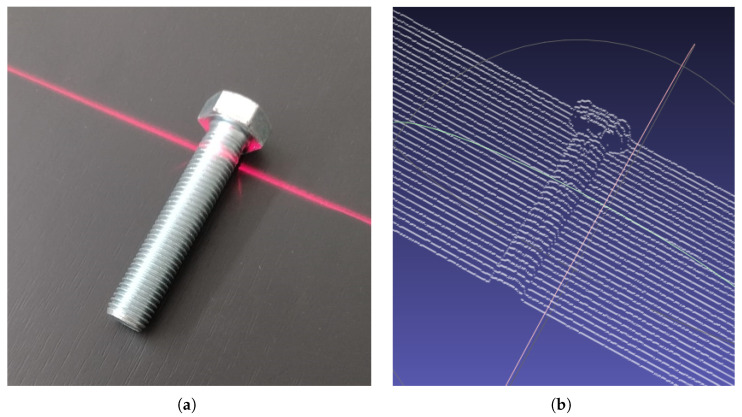
(**a**) The real bolt and (**b**) the point cloud resulted from the scanning process, visualized in MeshLab.

To further illustrate the applicability of the solution in an industrial context, the scanning was performed on a bolt, a common object in a pick-and-place operation, and the point cloud of the object is visible in Figure 25. The real height of the bolt at the end-side with the thread is 16 mm and at the head of the bolt it is 24 mm; the length is 85 mm; and the width at the thread is 15.5 mm, while the width at the head is 27 mm. The values obtained through the scanning process are 16.002 mm at the threaded end and 23.62 mm at the head, the length is 83.7 mm, and the width at the thread is 15.02 mm, while the width at the head is 27.53 mm.

The resulting precision and object point cloud demonstrate the utility of this type of scanner in industrial applications. Even though the bolt thread could not be precisely scanned, only the shape, position and orientation of the objects are needed in bin-picking applications, and with the resulting scanner resolution and precision, the objects’ attributes are properly obtained.

It should be noted that due to the variable width of the laser line, the precision in determining the length and height of the object will suffer. For a precise detection of these two dimensions, the laser line must fall on the object in its entirety, and since this might not happen every time, a procedure to compensate for this behavior is recommended. When testing the proposed solution, only the scenarios where the laser line is reflected in its entirety by the object are kept, and thus, in the table, the length of the real object was adjusted to account for this.

## 7. Limitations and Future Directions for Improvement

Per our observation, for the proposed method, the camera-calibration step, together with a precise pixel-to-millimeter ratio, is a crucial prerequisite to ensure good results; otherwise, the errors can add up fast and impact the results significantly. From the results, it can be observed that the error does not vary linearly with the height of the object, which seems to be a result of the camera calibration method, which has to be improved in the near future, or have the camera replaced with a better-performing one altogether. A method to compensate for the width of the laser line is desired as well, in order to minimize the errors on the x-axis and z-axis, namely, the length and height of the object. Other sources of error are the ambient light, the color of the objects and the surface finishes; specifically, reflective surfaces affect the performance of the scanning procedure.

To increase the scanning speed, more processing power is recommended, as well as developing a new, more efficient algorithm, leveraging a double-threaded pipeline, in which one thread is used for grabbing images, and in parallel, the other thread processes the already captured images.

We noticed that mathematically, there are multiple ways of approaching the issue to obtain the coordinates for the points on the object, and we briefly explored the options before choosing the one presented here. For the scope of this paper, we tried to keep the mathematical aspects simple, but it is also possible to test different mathematical approaches and compare the results.

Additionally, it is possible to explore solutions with multiple cameras, and therefore more complex mathematics and more precise results; however, this should be achieved in a personalized manner depending on the specifics of the industrial environment where it needs to be implemented, the area monitored, and the budget.

## 8. Conclusions

Three-dimensional scanning allows precise and efficient digital representation of physical objects, essential for modern industries such as aerospace, automotive, and manufacturing, where minor discrepancies can cause defects and increase the costs.

In response to the issues related to the affordability of the well-established industrial 3D scanning solutions available on the market, in this paper, we propose an open-source 3D scanning solution that has the advantages of being low-cost, transparent, and flexible, so that it can further be adapted to specific use cases and needs.

The presented 3D scanning solution was tested on both regular and irregular 3D objects, yielding satisfactory results. The results are particularly valuable and promising for unstructured industrial environments with fixed cameras.

Compared to the solution presented in the *State of the Art* section [29], which provided relative errors between 5.01% and 9.39%, our solution performed slightly better, with relative errors varying from 0.76% to 6.82%, while it should also be noted that our metrics were obtained on raw point clouds, while their metrics were obtained on smoothed meshes.

In the future, we plan on improving the proposed system by further minimizing the errors and speeding up the scanning process, as well as integrating it in a bin-picking application and comparing it with an industrial solution.

## Figures and Tables

**Table 1:** Table containing the notations used throughout the paper and their meaning.

Notation	Meaning
*D*	The distance extracted from the image as the distance between the laser line that falls on the floor and the laser line that falls on the object, projected perpendicularly onto the floor.
*H*	The height of the object that needs to be determined.
θ	The angle of the laser line, reflected from the mirror.
*C*	The distance between the laser line that falls on the floor and the laser line that falls on the object, as seen by the camera.
$\theta_{o}$	The angle at which the camera sees the laser line that falls on the object.
$\theta_{f}$	The angle at which the camera sees the laser line that falls on the floor.
$A_{v}$	The vertical distance from the horizontal line in the middle of the image and the laser line.
$H_{cam}$	The height at which the camera is mounted. In this case, $H_{cam}$ is 350 mm.
$A_{h}$	The horizontal distance from the vertical line in the middle of the image and the laser line.
*y*	The coordinate of the current point on the y-axis.
$y_{floor}$	The coordinate on the y-axis of the current point projected onto the floor, as seen by the camera.
dy	The deviation of the point seen by the camera $y_{floor}$ from the real coordinate *y* and the correction that must be applied to $y_{floor}$ to obtain the real coordinate *y*.
*x*	The coordinate of the current point on the x-axis.
$x_{floor}$	The coordinate on the x-axis of the current point projected onto the floor, as seen by the camera.
dx	The deviation of the point seen by the camera $x_{floor}$ from the real coordinate *x* and the correction that must be applied to $x_{floor}$ to obtain the real coordinate *x*.

**Table 2:** Table containing the parameters and values for the implemented system.

Parameter	Value
$H_{cam}$	350 mm
Camera resolution	1000 × 1000 pixels
Pixel-to-mm ratio	0.27083 mm/pixel
Camera field of view	270.83 mm × 270.83 mm
Laser and mirror height	409 mm
Stepper motor number of steps	2048 at full stepping; 4096 at half stepping
Angular step size for the stepper motor	0.17578125 degrees
Number of laser lines for a full scan	100
Linearized mm per laser step at floor level	2.7083 mm
Distance between camera and mirror	96 mm

**Table 3:** Table presenting a comparison between the real dimensions and the ones obtained through scanning.

Dimensions	Box 1 Real	Box 1 Scanned	Box 2 Real	Box 2 Scanned	Box 3 Real	Box 3 Scanned	Box 4 Real	Box 4 Scanned
Minimum Height [mm]	35	30.79	52	44.89	72	67.09	87	82.32
Maximum Height [mm]	35	35.14	52	54.00	72	75.92	87	89.56
Mean Height [mm]	-	32.61	-	49.44	-	71.45	-	86.00
Height Variance [mm^2^]	-	0.43	-	1.47	-	1.11	-	1.27
Height Mean Error [mm]	-	−2.39	-	−2.56	-	−0.55	-	−1.00
Height SD [mm]	-	0.66	-	1.21	-	1.05	-	1.13
Height Relative Error [%]	-	6.82	-	4.92	-	0.76	-	1.15
Minimum Length [mm]	29	27.36	67	67.24	130	130.68	79	77.98
Maximum Length [mm]	29	27.64	67	67.77	130	131.96	79	78.57
Mean Length [mm]	-	27.52	-	67.52	-	131.26	-	78.26
Length Variance [mm^2^]	-	0.004	-	0.011	-	0.104	-	0.014
Length Mean Error [mm]	-	−1.48	-	0.52	-	1.26	-	−0.74
Length SD [mm]	-	0.063	-	0.105	-	0.32	-	0.12
Length Relative Error [%]	-	5.1	-	0.77	-	0.97	-	0.94
Minimum Width [mm]	35	34.09	78	76.89	91	91.68	87	84.67
Maximum Width [mm]	35	34.61	78	77.71	91	93.00	87	87.10
Mean Width [mm]	-	34.41	-	77.27	-	92.30	-	86.04
Width Variance [mm^2^]	-	0.017	-	0.056	-	0.102	-	0.44
Width Mean Error [mm]	-	−0.59	-	−0.73	-	1.30	-	−0.96
Width SD [mm]	-	0.13	-	0.23	-	0.34	-	0.66
Width Relative Error [%]	-	1.68	-	0.94	-	1.43	-	1.1

## Data Availability

Dataset available on request from the authors.

## Abbreviations

The following abbreviations are used in this manuscript:

3D	Three-Dimensional
2D	Two-Dimensional
1D	One-Dimensional
AV	Artificial Vision
VR	Virtual Reality
MoCAP	Motion Capture
CMM	Coordinate Measuring Machine
6G	Sixth Generation
FPS	Frames per Second
FDM	Fused Deposition Modeling
3DFS	3D Forensics
RGB	Red, Green and Blue
DC	Direct Current
USB	Universal Serial Bus
SD	Standard Deviation
